# Researching Multisystemic Resilience: A Sample Methodology

**DOI:** 10.3389/fpsyg.2020.607994

**Published:** 2021-01-12

**Authors:** Michael Ungar, Linda Theron, Kathleen Murphy, Philip Jefferies

**Affiliations:** ^1^Faculty of Health, School of Social Work, Dalhousie University, Halifax, NS, Canada; ^2^Department of Educational Psychology, Faculty of Education, University of Pretoria, Pretoria, South Africa

**Keywords:** resilience, multisystemic resilience, methodology, resilience across cultures, resilience in stressed environments

## Abstract

In contexts of exposure to atypical stress or adversity, individual and collective resilience refers to the process of sustaining wellbeing by leveraging biological, psychological, social and environmental protective and promotive factors and processes (PPFPs). This multisystemic understanding of resilience is generating significant interest but has been difficult to operationalize in psychological research where studies tend to address only one or two systems at a time, often with a primary focus on individual coping strategies. We show how multiple systems implicated in human resilience can be researched in the same study using a longitudinal, six-phase transformative sequential mixed methods study of 14- to 24-year-olds and their elders in two communities dependent on oil and gas industries (Drayton Valley, Canada, and Secunda/eMbalenhle, South Africa). Data collection occurred over a 5-year period, and included: (1) community engagement and the identification of youth health and well-being priorities; (2) participatory youth-centric qualitative research using one-on-one semi-structured interviews and arts-based methods; (3) survey of 500 youth at three time points to assess psychosocial health indicators and outcomes; (4) collection of hair samples to assess stress biomarkers (cortisol and dehydroepiandrosterone-DHEA) over time; (5) youth-led ecological data collection and assessment of historical socio-economic development data; and (6) community resource mapping with community elders. Analyzing data from these multiple systems will allow us to understand the interrelationship and impact of PPFPs within and across systems. To date, we have undertaken thematic and narrative qualitative analyses, and descriptive analyses of the preliminary ecological and survey data. As we proceed, we will combine these and grounded theory approaches with innovative techniques such as latent transition analysis and network analysis, as well as modeling of economic conditions and spatial analysis of human geographies to understand patterns of PPFPs and their inter-relationships. By analyzing the complexity of data collected across systems (including cultural contexts) we are demonstrating the possibility of conducting multisystemic resilience research which expands the way psychological research accounts for positive development under stress in different contexts. This comprehensive examination of resilience may offer an example of how the study of resilience can inform socially and contextually relevant interventions and policies.

## Introduction

While human resilience was once conceptualized as a set of individual traits that predisposed children to successful coping under stress (Block and Block, 1980; Block and Kremen, 1996; Kumpfer, 2002), momentum has been growing to understand resilience as a process that includes interactions occurring within and between multiple systems, ranging from individual biology to psychological, relational, sociocultural, institutional and ecological mechanisms that create the potential for populations under stress to do better than expected (Rutter, 1987; Masten et al., 1990; Egeland et al., 1993; Werner, 1993; Masten and Cicchetti, 2015; Ungar and Theron, 2020). The increasing attention toward interacting systems of influence has shifted focus from individual disorder and dysfunction in the psychological and social sciences, to the positive impact of promotive and protective factors and processes (PPFPs) that facilitate resilience in contexts of exposure to atypical stress or adversity. When defined multisystemically (Ungar, 2020), resilience is not merely the product of one aspect of an individual’s life (Infurna and Luthar, 2018), but rather is facilitated by multiple PPFPs at multiple systemic levels (Ungar and Theron, 2020). Like other frameworks for positive development (e.g., Positive Youth Development—(Lerner et al., 2013); Positive deviance—(Marsh et al., 2004); Positive Psychology—(Seligman and Csikszentmihalyi, 2000), designing and implementing high-quality research to understand complex systems and the interactions associated with resilience has been difficult to accomplish. In the absence of methodological guidebooks, studies of human resilience have tended to address only one or two systems at a time, often with a primary focus on individual and family-level resilience factors (Kumpfer, 2002; Fritz et al., 2018a). While useful, these studies have an implicit bias, suggesting that resilience is affected most by individual adaptation to a stressful environment. This is despite the recognition of influences at the meso- (i.e., interactions between individual factors), exo- (i.e., the institutional environment within which one operates), and macrosystem environments (i.e., culture, policy, law) that shape the resilience of individuals (Ozbay et al., 2010; Hobfoll et al., 2011; Wang et al., 2014), in ways reminiscent of Bronfenbrenner’s Ecological Systems Theory (Bronfenbrenner, 1989).

The study design discussed in this paper is an illustration of how to operationalize resilience research that accounts for multiple PPFPs across different systemic levels at the same time. The *Resilient Youth in Stressed Environments* project applies Ungar and Theron’s (2020) systemic model of resilience to a complex social ecology. Specifically, the design uses a 6-phase transformative sequential longitudinal and mixed methods study to examine the lives of 14- to 24-year-olds and their elders in two communities dependent on oil and gas industries and susceptible to boom-and-bust economic cycles: Drayton Valley, Canada, and Secunda/eMbalenhle, South Africa. By analyzing the complexity of data collected across systemic levels and in two diverse sociocultural contexts, we are demonstrating the possibility of conducting multisystemic resilience research that expands the way psychological research explains positive development under stress.

### Background

The term resilience appears in disciplines as diverse as epigenetics, psychology, disaster risk reduction, environmental science, public health, and economics. Most typically, ‘resilience’ is used to describe how a system of any size (whether a traumatized brain, a refugee family, a community destroyed by fire, or a coral reef bleached by agricultural runoff) not only recovers from adversity, but manages to sustain itself and thrive. Across all these disciplines, the goal has been the same: to shift our focus from why things go wrong in response to some adverse event or circumstance to the factors and processes that protect individuals and systems from breaking down.

Yet comprehensive resilience research is difficult to operationalize in practice for several reasons. First, the concept of resilience has been somewhat ambiguous in the literature to date, with no shared definition that reflects its complexity (Southwick et al., 2014), much less its multisystemic influences (Ungar, 2020). Resilience, for instance, has been discussed as both an individual attribute, such as the concept of ego-resiliency by Block and Block (1980), and as a product of self-esteem and self-efficacy by Rutter (1985). In other cases, resilience is described as a process, as in work by Luthar et al. (2000) where it is defined as “a dynamic process encompassing positive adaptation within the context of significant adversity” (p. 1). Further still, the ecological-transactional model of community violence and child maltreatment (Cicchetti and Lynch, 1993) outlines various systemic levels (i.e., ranging from the ontogenic development of an individual to the exosystemic factors that shape an individual’s environment) within which protective factors can facilitate the successful adaptation of young people experiencing violence. Building on systems theories of resilience, Curtis and Cicchetti (2003) also convey the importance of considering human biology (e.g., neuroendocrinology, neuroplasticity, genetics, emotional regulation, etc.) as encompassing domains of influence on an individual’s resilience. Together, these definitions have entered into what several scholars have termed a ‘fourth’ wave of resilience research (Masten, 2007, 2011; Doty, 2010; Wright et al., 2013), where the focus is on multilevel and integrated analysis across biological, psychological, and environmental systems.

Along with the challenges of conceptualizing resilience as a construct, it is difficult to operationalize resilience when designing empirical research (Cicchetti and Garmezy, 1993; Kaufman et al., 1994; Luthar et al., 2000; Stouthamer-Loeber et al., 2002; Cumming et al., 2005; Doty, 2010). Resilience is oftentimes measured using risk and protective factors (Fergus and Zimmerman, 2005; Doty, 2010; Ungar, 2019), yet tensions exist with how to distinguish between the two (Luthar and D’avanzo, 1999). For instance, a particular factor like emotional withdrawal may be protective for one individual in a context of constant abuse by a caregiver but pose a risk to development for another where secure and safe attachment with a caregiver is possible. In this regard, differing fields of thought exist as to whether resilience can be objectively measured, or if one’s subjective perception of their risk and protective factors governs their resilience (Jones and Tanner, 2015; Jones, 2019).

The protective value of any given process or factor is also sensitive to contextual and cultural dynamics. For instance, the resilience of Black South African adolescents is positively influenced by receiving care from strong Black women, whether or not those women are biologically related to the youth (Ungar and Theron, 2020); in contrast, studies of youth resilience in typically Western contexts point to the salience of primary caregivers (Masten, 2015). A consideration of such contextual and cultural determinants of resilience have been largely omitted from the resilience literature despite acknowledgment of their influence on PPFPs (Feldman and Masalha, 2007; Ungar, 2008, 2019; Raghavan and Sandanapitchai, 2019; Ungar and Theron, 2020). To overcome these issues and produce more contextually sensitive resilience research, study designs need to assess “(1) the quality and quantity of risk exposure (with greater contextual sensitivity), (2) the PPFPs that interact at biological, psychological, social, economic and ecological levels, and (3) the many possible outcomes that are associated with recovery, adaptation and transformation (and why some outcomes are privileged as more desirable than others)” (Ungar, 2019, p. 2). When these steps are taken, researchers are likely to be better positioned to avoid reinforcing neo-liberal bias which presupposes homogeneous experience of stress across populations and individual responsibility to thrive when facing adversity (Joseph, 2013; Liebenberg et al., 2015; Garrett, 2016). A more contextualized, multisystemic reading of resilience suggests that populations are diverse in which factors are most useful for maintaining wellbeing and the shared responsibility for individual success.

## Methods

### Research Context

Resource extraction communities attract both temporary and long-term workers to capitalize on economic opportunities (Brown et al., 2005). However, dependence on a single industry leaves these communities vulnerable to dramatic fluctuations in the price of commodities, often described as boom and bust cycles (Tokic, 2015; Mohaddes and Pesaran, 2017). During economic downturns (i.e., ‘busts’), communities experience spikes in unemployment and poverty (Marchand, 2012; Jacobsen and Parker, 2016), out-migration of short- and long-term residents, a reduction in community support to vulnerable populations (Graves et al., 2009; Van Assche et al., 2017), and an increase in mental health challenges among community members (McClelland, 2000; Frasquilho et al., 2016; Virtanen et al., 2016). Economic ‘booms’ can also pose challenges to resource extraction communities, such as the rapid influx of workers overburdening community services (Schafft and Biddle, 2015; McLaughlin et al., 2017), an increase in family separation resulting from excessively long work hours (Markey et al., 2015), youth leaving school early to enter the industry (Schafft and Biddle, 2015; Von Simson, 2015), and an increase in substance use and crime (Luthra et al., 2007; Ruddell, 2011; Ruddell and Ortiz, 2015). Because of the many stressors resulting from the volatility of resource-dependent economies, and our lack of knowledge of how young people navigate their way through these challenges, we have focused our study on the resilience of youth in two communities dependent on oil and gas extraction and processing industries: Drayton Valley, Canada, and Secunda/eMbalenhle, South Africa. Together, these two communities provide settings to investigate the heterogeneity in the factors and processes associated with resilience in both the Global South and the Global North.

**FIGURE 1:**
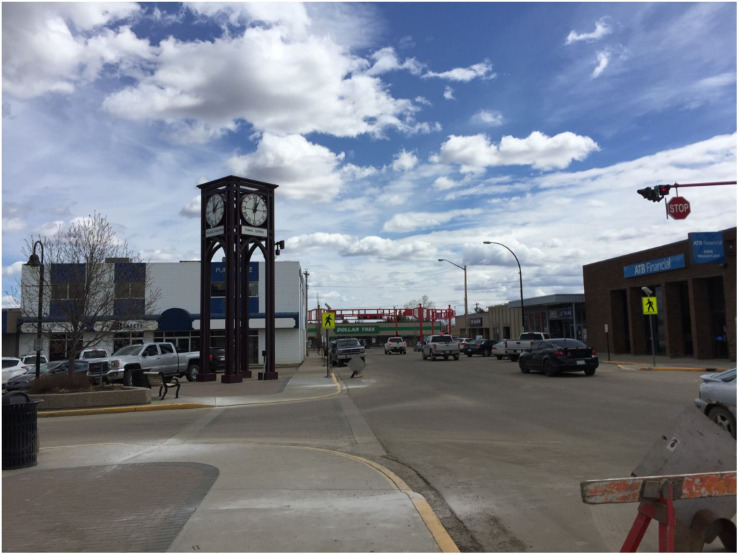
Photo of the town of Drayton Valley, taken by a member of the research team.

Drayton Valley, 133 km southwest of Edmonton, Alberta (the largest oil-producing Province in Canada), was established in 1953 (Figure 1). Home to approximately 7,000 people, it is largely dependent on oil and gas extraction, agricultural, and forestry, although most of the workforce is employed by the oil and gas industry (Ungar and Theron, 2020, p. 3). Drayton Valley experienced five boom and bust periods since 1996, with the most current enduring since 2014. During this same period, there have been decreases in the quality of the natural environment, with fewer hectares of waterways, wetlands or barren land (e.g., unused open areas), and forest area cover, along with concurrent increases in the amount of land being converted to agriculture.

**FIGURE 2:**
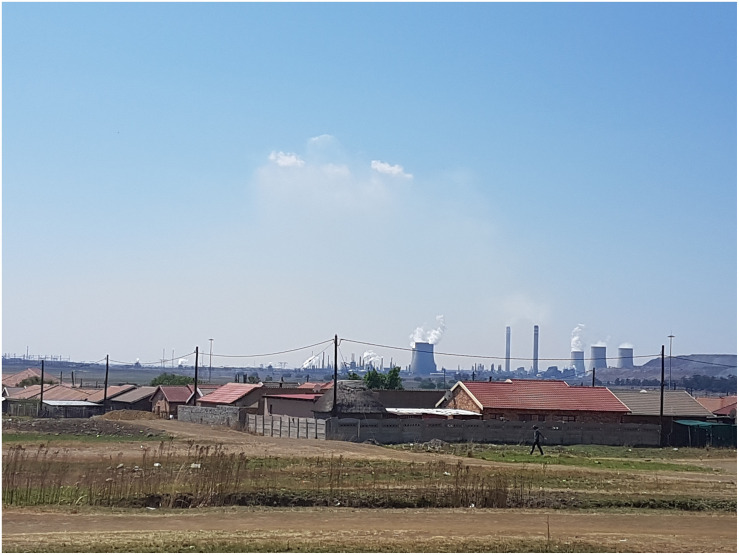
Photo taken by the research team of the township of eMbalenhle and the neighbouring coal liquification plant.

The second community, eMbalenhle is a small township proximate to the more developed town of Secunda, located approximately 150 km East of Johannesburg, South Africa (Figure 2). Secunda/eMbalenhle are located in Mpumalanga, one of South Africa’s poorer provinces. It is home to the largest underground coal-mining complex in the world (Govan Mbeki Municipality, 2019), as well as a coal liquefaction plant which produces synthetic fuel, petroleum, paraffin, jet fuel, creosote, bitumen, diesel, and lubricants. Over the past decade, this plant has been identified as a significant emitter of CO_2_, while also being recognized for continual job creation opportunities and for investing in the community (Mondliwa and Roberts, 2019). In 2011, the population of Secunda was approximately 40,000 people, with roughly 250,000 more living in the wider Govan Mbeki municipal area (Statistics South Africa, 2011). Of these, approximately 100,000 reside in the township of eMbalenhle. The unemployment rate in the wider municipality is 26%, and 34.4% among youth aged 15 to 34 (Statistics South Africa, 2011). In 2016, only 44.4% of households in the municipality as a whole (which includes eMbalenhle) had access to in-home piped water (Statistics South Africa, 2016).

### Study Participants

Young people aged 13–24 from Drayton Valley and Secunda/eMbalenhle were invited to participate, as this age group is more likely to be affected by changes to their communities and environments, and are less protected from external stressors by their families of origin during this developmental period (de Jong et al., 2015). Local advisory committees (LACs) were established in each community, comprising key stakeholders drawn from youth services, schools, oil and gas industries, and government departments, along with young people themselves and members of the research team when appropriate. The LACs have not only advised the project on the appropriateness of the research methods in each context, but aided in locating young people that fit the study’s inclusion criteria, ranging from those doing better than expected to those struggling to cope in an unpredictable environment. Specifically, we required cohorts of youth who have been exposed to economic, social, or environmental disruptions (positive and negative) related to the oil and gas industry. As those young people who have encountered particularly difficult social and economic challenges may be less willing and able to participate (because, for example, they are unable to take the time to do so, they are precariously housed and therefore difficult to locate, etc.), our LACs played an important role in inviting them to the study, given their understanding of their community and their network of community members. Adults over the age of 24 were also included for Phase 6 of the study. Each sample included approximately equal numbers of females and males to explore sex and gender-specific patterns of resilience.

To locate different SES groups, we drew youth from school districts that vary by high and low SES. In South Africa, all schools are nationally ranked into quintiles based on the vulnerability of the students (Hall and Giese, 2009). In Drayton Valley there are multiple high schools, however, the economic status of students varies within each school district and fluctuates based on the price of oil and gas. To deal with these challenges, we diversified the sample by selecting students in school-based feeding programs, and youth in alternative education settings. To locate youth who are not in high school or post-secondary education, and employed youth, LACs advised a number of snowball, purposive, and convenience sampling techniques such as hosting social events, setting up data collection sites next to stores and businesses frequented by young adults, and door to door surveys in neighborhoods where many youth are likely to reside. Half-way through sampling, we conducted *post hoc* comparisons to each community’s demographic profile to see how the sample compares and if more targeted recruitment was required. Participants in both sites were reimbursed for their participation in each phase of data collection.

### Study Design and Timing

**FIGURE 3:**
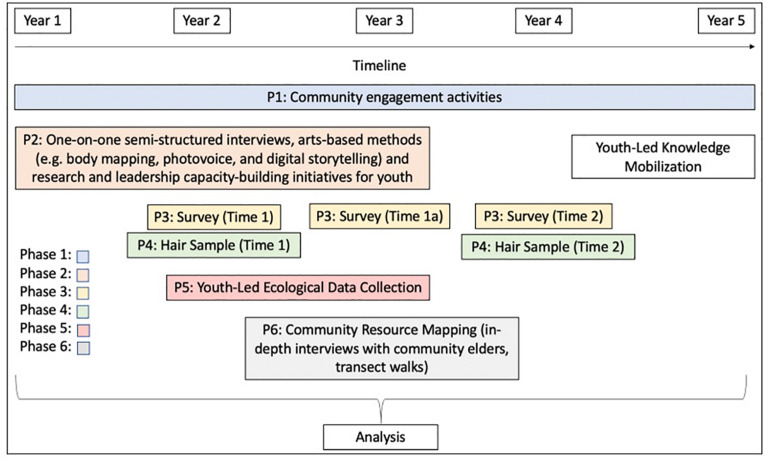
Timeline for data collection and analysis activities for RYSE: Canadian and South African sites.

The Resilient Youth in Stressed Environments (RYSE) project examines the interactions between the biological and psychological resilience of young people, community resilience, and the resilience of environmental systems. Transgressing disciplinary silos, this research uses a transformative sequential (i.e., occurring in sequence) mixed methods design to facilitate data collection across multiple human and ecological systems. The transformative paradigm offered the ontological, epistemological, methodological, and axiological foundations to ensure a respect for the role of power differentials in shaping participant realities and the relationship between researchers and community members, and the need to uphold social justice and privilege community voices at each stage of the project (Mertens, 2016). The six phases of the project were informed and guided by LACs in each community, beginning prior to the project’s inception when key stakeholders requested resilience research be undertaken in their communities. Members of each LAC were involved at the grant writing stage, and met regularly throughout each phase of the project to (a) ensure the research was appropriate (e.g., protecting the over-researching of their communities), (b) suggest culturally and contextually relevant data collection tools and approaches (e.g., requesting specific questions of relevance to the community, suggesting how best to collect the data so as not to overburden or inconvenience participants, etc.), (c) assist with youth recruitment, (d) contribute to data analysis by suggesting what to look for in the data and validating findings, and (e) brainstorm and assist with youth engagement and knowledge mobilization strategies and activities. Such tasks have been well documented in the literature on the role of community advisory committees in research (Koné et al., 2000; Reddy et al., 2010; Nyirenda et al., 2018; Mlambo et al., 2019), and in resilience research specifically (Theron, 2013; McCubbin and Moniz, 2015). We also maintained open channels of communication with the LACs, as well as the communities at large (e.g., through school board and municipal council meetings, as well as open meetings for anyone interested in attending), to make space for iterative and ongoing feedback and discussion throughout the project’s lifecycle. This type of meaningful community engagement has been recognized for protecting participants and ensuring the research is acceptable to the community, building community-researcher partnerships, and strengthening the quality of the research (Mlambo et al., 2019). Each of the six phases of the research is described below, and the timeline for the project is shown in Figure 3.

#### Phase 1: Community Engagement

Two community engagement workshops (one with youth and one with adults) were held in each community. Community engagement is an important first step within our transformative paradigm, and any participatory research, in order to build trusting relationships with community members, and to ensure the data collected aligns with community needs. While there is little guidance as to how to do community engagement (Shalowitz et al., 2009), we decided to host community workshops to provide a relaxed yet structured environment for building relationships and understanding community needs. Youth and adults were recruited using purposive sampling with the support of the LACs, and by promoting the workshops on social media platforms. These workshops engaged youth between the ages of 13 and 24 (*n* = 13 in Canada; *n* = 6 in South Africa) and adults over the age of 24 (*n* = 15 in Canada; *n* = 4 in South Africa) with the goal of identifying youth priorities related to community resilience, health and wellbeing, and strengths and challenges associated with community resilience in boom and bust economies. They included facilitated discussions; community mapping exercises; relationship-building activities such as a bowling pizza event at the Canadian site and a working breakfast with youth in South Africa; and information on Phase 2 youth research activities. Field notes, group discussions and visual content from interactive activities were captured and transcribed for analysis. The content of these workshops was used to guide the introduction of the research to the community and develop an interview guide for the more structured meetings with youth during Phase 2. From these initial engagement workshops, we also established Youth Advisory Committees in each community to help facilitate youth engagement throughout the project’s lifecycle, and to inform and lead research and knowledge mobilization activities.

#### Phase 2: Participatory Youth Research

Data were collected from youth through one-on-one semi-structured interviews (*n* = 45 in Canada; *n* = 21 in South Africa), joint interviews (*n* = 6, for Canada only), and focus groups (*n* = 11, for South Africa only) (all of which lasted approximately 40–60 min), and participatory arts-based methods such as photovoice (*n* = 8 in Canada; *n* = 6 in South Africa) (Wang and Burris, 1997; Budig et al., 2018), digital storytelling (*n* = 8 in Canada; *n* = 16 in South Africa) (Gubrium, 2009; De Vecchi et al., 2016), body mapping (*n* = 30 in South Africa, in South Africa only) (Gastaldo et al., 2018), and ‘Draw, Write, and Talk’ (*n* = 30, in South Africa only) (Mitchell et al., 2011; Angell et al., 2014; Machenjedze et al., 2019). As part of the Participatory Action Research methodology, youth were also trained by research staff to conduct peer-to-peer interviews (*n* = 8 in Canada; *n* = 12 in South Africa) through research and leadership capacity-building workshops. Interviews and focus groups with youth covered topics such as health and well-being priorities, coping strategies, family and community support, and the impact of the boom and bust cycle associated with the oil and gas industry. Youth were recruited through nominations by the LACs, community partner organizations, and other youth services within each community. Staff first sought the permission of youth to have them nominated, and then a researcher followed up to confirm their participation. Snowball sampling was also utilized, where participants recommended the study to their peers, as well as placing recruitment posters throughout the town and on social media. Written or verbal consent was obtained from youth prior to participation. For those youth under the age of 16, consent was obtained from their parents, and assent was obtained from the youth. All data collection activities were recorded and transcribed verbatim. Transcripts were de-identified and inputted into Atlas-ti software for analysis.

#### Phases 3: Youth Surveys

A survey was developed and piloted with a small number of youth (*n* = 6 in Canada; *n* = 6 in South Africa) who were part of the advisory committee or previous participants in Phase 1 and 2 activities. Survey items were modified based on suggested additions and deletions, and surveys were implemented in years two (T_1_) and four (T_2_). T_1_ and T_2_ surveys were administered in schools and community centers by community-based research assistants and members of the research team. A T_1_a survey (a slightly abbreviated version of T_1_) was administered online using Opinio software for the Canadian site only. In the Canadian site, 500 participants took part in T_1_, 294 in T_1_a, and 306 in T_2_. In the South African site, 572 participants took part in T_1_, and 349 in T_2_.

Participants were recruited via promotion of the project over social media and posters distributed throughout the community, classroom presentations to sensitize youth to the research, referrals by service providers and LACs (with the permission of youth), and by youth sharing the opportunity with their peers (i.e., snowball sampling). Participants were contacted periodically between surveys to keep them engaged and update their personal information.

#### Phase 4: Youth Hair Samples

To track changes over time, we collected hair samples at baseline (*n* = 399 in Canada; *n* = 431 in South Africa) and 24 months (*n* = 278 in Canada; *n* = 284 in South Africa) alongside survey administration. As such, participants were recruited through the same means as in Phase 3. A trained community researcher, as well as members of the research team, retrieved hair samples from youth. Where participants had to mail their hair samples (in Canada only), detailed instructions were provided, including access to a short video to explain the procedure. Once received by the research team, samples were mailed in batches of 15 (to avoid large losses) to the respective Canadian and South African labs. Hair cortisol and DHEA will be analyzed using kits obtained from Salimetrics, LLC, State College, PA, United States (High Sensitivity [Salivary] Cortisol Immunoassay Kit, Cat# 1-3002 and High Sensitivity [Salivary] DHEA Immunoassay Kit, Cat# 1-2212) [Please note: These kits were originally designed to measure cortisol and DHEA in saliva but can measure these hormones from any source (Vaghri et al., 2013)]. Analyses from the South African and Canadian labs were validated by comparing the results of a subset of samples analyzed in each lab.

#### Phase 5: Citizen Scientists

To examine indicators of ecological systems and their interactions with humans (Folke et al., 2016), we engaged young people in an intensive 9-day workshop in Canada and a 4-day workshop in South Africa. Youth ages 18–24 were recruited for this workshop (*n* = 10 in Canada; *n* = 12 in South Africa) by sharing the opportunity through the LAC and on social media. The research team and LACs assessed the written applications of interested youth. The higher age range was required due to drone use licensing (in Canada) and ethical considerations. The length of each workshop was based on youth availability. As well, while the Canadian youth received training and certification in the use of drones, no such training was available as part of these workshops for youth in South Africa. During each workshop, the youth participated in six different participatory research activities, which as a whole identified a place-based narrative of youth experiences and perceptions of social-ecological system change. These six activities included: (1) the Q methodology, (2) asset mapping, (3) Citizen Scientist Survey 123, (4) participatory mapping, (5) ecological monitoring, and (6) the use of Unmanned Arial Vehicles (UAVs), or drones, for youth to capture spatial images of their communities.

#### Phase 6: Adult Interviews and Transect Walks

This phase explored the personal lives and adaptation patterns of adults 30 years or older in both communities as well as their perspective of their community’s risks, resilience resources, and historical development. The contribution of adult informants helped the research team understand temporal dimensions of changing systemic interactions and the broader social ecologies that shape young people’s experiences. Face-to-face semi-structured interviews were conducted in order to assess theoretically relevant factors based on the preceding phases of the RYSE project, as well as give room for exploration of new patterns (*n* = 37 in Canada; *n* = 31 in South Africa). Recruitment for adult interviews took place through snowball sampling, referrals from the LAC and via social media platforms. Those participants nominated to the study by the LACs were chosen based on length of time in community and diversity of social location (e.g., economic status, race, sex, occupation). The interviews lasted 60–90 min, and all interviews were audiotaped and transcribed for the analysis. For the transect walks, we purposively sampled a subpopulation of participants who had completed the adult interviews based on the heterogeneity of experience they could introduce to the data (*n* = 16 in Canada; *n* = 11 in South Africa).

### Data Collection

#### Biological Data

When we experience stress, our body mobilizes adaptive responses to remove the stressor and restore homeostasis (Johnson et al., 1992; Chrousos, 2009) which contributes to resilience. Confrontation with a stressor, whether physical or psychological, causes activation of the sympathetic nervous system which acts on the adrenal medulla (central portion) resulting in the release of adrenaline and a “fight or flight” response. When the stress response system is activated, a cascade of hormonal responses also results in the release of the glucocorticoid hormone cortisol, the major stress hormone in humans, from the adrenal cortex (outer part). Chronic exposure to adverse experiences, including stressed environments, can have negative developmental and health consequences over the life course (Hertzman, 2013) becoming biologically embedded into the molecular, physiological, neurobiological, and genomic systems that determine our level of vulnerability and resilience (Boyce et al., 2012). Both family environments (e.g., mother’s experience of stress or depression) (Lupien et al., 2001; Essex et al., 2002) and socioeconomic status (SES) (Lupien et al., 2000; Li et al., 2007) influence cortisol secretion patterns. Recently, methods to assay cortisol in hair have been developed which provide a complementary means of monitoring stress. Increasing evidence suggests that hair cortisol levels provide an integrated index of cumulative cortisol exposure over an extended period of time and thus measures of hair cortisol show promise for the investigation of relationships among social and environmental factors, stress, life-course events, and biological activity related to stress (Russell et al., 2012).

In addition to cortisol, we are measuring DHEA (dehydroepiandrosterone), another hormone secreted by the adrenal cortex in response to stress. DHEA is classified as a neurosteroid (i.e., it is produced in the brain as well as the adrenal gland), and thus can affect central nervous system function, including behavior. Importantly, DHEA has anti-glucocorticoid (cortisol) effects and thus may protect the body from high levels of cortisol (Charney, 2004) and from the detrimental effects of stress (Kalimi et al., 1994; Kaminska et al., 2000). Data suggest that morning and afternoon DHEA levels are related to resilient functioning; for example, maltreated children who show high degrees of resilience were reported to have an unexpected rise in DHEA across the day (Cicchetti and Rogosch, 2007), which would result in a low cortisol/DHEA ratio (i.e., DHEA has increased relative to cortisol and can thus exert its anti-cortisol effects), and thus better regulation of the stress response system. By contrast, a high cortisol/DHEA ratio may be suggestive of a greater or more chronic stress response and thus reduced resilience. It has been suggested that because of the antagonist action of DHEA to cortisol, measurement of cortisol alone may provide an incomplete estimate of hypercortisolemia or chronically elevated cortisol levels (Goodyer et al., 1998), and that a more sensitive measure of the degree of “functional” hypercortisolemia is by calculation of the cortisol/DHEA ratio. DHEA, like cortisol, can be measured in hair.

#### Psychosocial Data

The PPFPs commonly linked to positive outcomes under significant stress include healthy attachment, self-regulation, agency and mastery, problem-solving and meaning-making (Masten and Wright, 2010). Each process draws on resources within the individual (e.g., social skill, tenacity, intelligence), as well as resources within the social ecology in which the individual is nested (e.g., supportive mentors, educational opportunity, nutritious food) (Ungar, 2012; Rutter, 2013; Masten, 2014). In this sense, psychological resilience is an interactive and social process in which individual coping capacity is triggered through facilitative environments that make it possible to experience psychological wellbeing despite exposure to chronic or acute adversity, including mental disorders (Masten, 2001; Bonanno and Mancini, 2012). Depending on various factors (e.g., the intensity of risk encountered, one’s access to services and supports, etc.), some processes (and the resources they draw upon) matter more – or less –to the achievement and maintenance of functional outcomes (Schwerdtfeger Gallus et al., 2015), and they can look different depending on one’s sociocultural and historical context (Masten, 2014; Theron and Liebenberg, 2015).

Psychological resilience was studied first through qualitative interviews and focus groups, in some instances using arts-based methods, as well as through a longitudinal survey administered at three timepoints over 24 months. Survey items were drawn from an existing measure of adolescent risk and resilience previously administered by the principal investigators to over 7,000 young people and caregivers in South Africa, New Zealand, China, Colombia and Indigenous and non-Indigenous communities in Canada. The survey for Time 1 (T_1_) comprised of a total of 219 items in the Canadian survey, and 235 items in the South African survey. These were divided into four broad domains (demographics, risks, PPFPs, and outcomes), with the recognition that there is fluidity between them (e.g., in some cases a demographic variable may also be considered a risk, etc.). The LAC in each community provided guidance on modifying and omitting questions where necessary to suit the local contexts, as well as creating new questions for the purpose of this project.

The demographic questions included items shared across both surveys regarding the race of the respondent, their sex, age, languages spoken, time spent in the community, and their school and work status. All demographic questions were created specifically for this survey, save for one item from Statistics Canada (Government of Canada, 2017), “What race do you identify with?,” and one item in the Canadian survey on sexual identity recommended by The Fenway Institute (2017), “Do you think of yourself as… lesbian, gay, homosexual/straight or heterosexual/bisexual/don’t know/something else.” This item was modified slightly by replacing the responses of ‘something else’ with ‘other’ and including a ‘prefer not to answer’ option. It was not included in the South Africa survey, as the LAC voted against its inclusion given the prevalence of homophobia in the target community. Additional questions asked in the South Africa survey included, “what type of house do you live in most of the time?,” “what is the main source of water at your everyday home?,” “what type of toilet do you use at your everyday home”? and other such questions that offer insight into the contextually relevant markers of disadvantage.

The risk domain included items drawn from the Child Post-Traumatic Stress Reaction Index (CPTS-RI; Pynoos et al., 1987; Frederick et al., 1992; Nader et al., 1993) (adapted for non-students/older youth; example internal consistency from the Canadian site: α = 0.91. All alpha coefficients for subsequent scales are examples from the Canadian site); the Impairment Associated with the Traumatic Symptoms Scale (adapted slightly for readability; α = 0.84) (Ruchkin et al., 2004); the Short Form Health Survey (SF-20; Stewart et al., 1988) (which omitted the mental health subscale as we measured depression via the Beck Depression Inventory-II elsewhere; α = 0.88); the Victimization by Community subscale of the Exposure to Violence scale (in the Canadian survey we removed “attacked or stabbed with a knife,” “shot or shot at with a gun,” and “threatened or harmed by someone because of my race or ethnicity” because we felt that they were largely addressed by other items in this scale; α = 0.82) (Richters and Martinez, 1993; Ruchkin et al., 2004), the Family Adversity scale (adapted from Labella et al., 2017) (omitting from the Canadian survey the item about time being separated from parents, changing the wording to be more understandable to youth, and changing the survey to a self-report rather than being completed by parents; α = 0.73), the Perception of Neighborhood scale (Ruchkin et al., 2004) (with slight modifications; α = 80), and finally, questions created specifically for this survey that address perception of community spaces, and parent and household status.

The PPFPs domain included the Child and Youth Resilience Measure (CYRM-28; Ungar and Liebenberg, 2011; Liebenberg et al., 2012). The three CYRM-28 sub-scales assess (1) individual resources including personal skills (such as ability to problem solve, cooperation, and awareness of personal strengths), peer support, and social skills (α = 0.87); (2) relationships with parents or primary caregivers including physical and psychological caregiving (α = 0.87); and (3) contextual resources that facilitate connection to culture, the role of religious and spiritual beliefs, and engagement with and relevance of education (α = 0.82). We also used the Benevolent Childhood Experiences scale (Narayan et al., 2015) which has been used to complement the study of Adverse Childhood Experiences (α = 0.68), and we measured sensitivity through the 6-item Sensitivity scale (very short version; Pluess, personal communication; α = 0.63). In the South Africa survey, parental supervision and parental warmth were measured using 4-item subscales from Parenting Scale (Ruchkin et al., 2004).

The outcomes domain focused on school and workplace engagement, depression, peer group support, and risky behaviors. These items were drawn from the School Engagement Scale (Lam et al., 2014) (α = 0.94); a new question probing perception of grades/marks in the Canadian survey; the Peer Support Scale (short) (Armsden and Greenberg, 1987; Lerner et al., 2005) (α = 0.93), a delinquency scale (Geldhof et al., 2014) with an additional question on bullying (α = 0.82), the Beck Depression Inventory (BDI-II; Beck et al., 1996) (α = 0.95), the Work Engagement Scale shortened (Schaufeli and Bakker, 2004; Schaufeli et al., 2006) (α = 0.96), and the Substance Abuse scale for the Canadian survey only (α = 0.75–0.86 across grades 7 through 12) (Geldhof et al., 2014). In the Canadian survey only, additional questions were added specifically for this project to inquire about unprotected sexual intercourse, the best things about the community, and social media use and how individuals learn about news in their community.

For the final administration of the survey (T_2_), several new questions were added to the Canadian survey, including assessments of leisure time, access to transportation, financial management, financial knowledge, and whether the participant had moved since completing the first survey. Stress was also measured using the 10-item Perceived Stress Scale (Cohen et al., 1983) to compare with the hair cortisol and DHEA levels; coping was measured through the 28 items in the Brief COPE (Carver, 1997), which captures 14 overarching coping styles; and device use/screen time was measured using an adaptation of the Screen Time Questionnaire (Vizcaino et al., 2019).

#### Community Data

For purposes of this study, we defined a community as being the geographically bordered and socially coherent space where people interact and receive services. Community resilience draws on the perspectives of multiple disciplines to help frame an understanding of how human biological and psychological systems, along with people’s physical and social ecologies dynamically influence each other to create vulnerabilities, adaptive capacities, and shared resilience (Cox, 2015; Cox and Hamlen, 2015). This view of resilience implies a broad range of interconnected social, psychological, cultural, and structural factors that influence local capacity to anticipate, adapt to, and weather shocks, such as disasters, climate change, and other catastrophic environmental, economic and socio-political changes (Folke, 2006; Gotts, 2007).

In the context of community resilience, this study’s analytical focus was on understanding social vulnerability which refers to the “characteristics and circumstances of a community, system or asset that make it susceptible to the damaging effects of a hazard” (UNISDR, 2009, p. 30). In other words, social vulnerability results from social conditions and circumstances (present day and historic) that are related to individual biological and psychological characteristics (e.g., mental health, income, gender, ability, class, developmental stage and age, education, race, and ethnicity) but it is not these individual characteristics in and of themselves that create vulnerability. It is, rather, the ways in which society and/or communities recognize and respond to these characteristics that creates social and structural vulnerabilities (Prilleltensky and Prilleltensky, 2007). Furthermore, social vulnerability and resilience are understood to be at least partially socially constructed, reflecting the differential distribution of resources and the ways in which individual characteristics (e.g., gender, age, ability, ethnicity) intersect with structural vulnerabilities (e.g., poverty, land use decisions) and strengths/resources to create variable patterns of vulnerability and resilience (Adger, 2006; Fordham et al., 2013; de Jong et al., 2015; Ungar, 2015). Community resilience is, therefore, a strengths-based construct, focusing on capacities and assets and how these can be mobilized and/or enhanced in order to reduce community vulnerability and risk and promote community transformation (Cox, 2007; Archibald and Munn-Venn, 2008; Cutter et al., 2008a, b; Folke et al., 2010).

To assess community resilience we conducted transect walks, whereby researchers and a group of knowledgeable community members were to walk along a pre-defined path (a transect) in order to identify and map community-based environmental resources and/or deficits, as well as sociocultural resources (Hemmersam and Morrison, 2016). Specifically, we were interested in generating a sense of historical community resilience related to the boom and bust periods and identifying spaces within each community that offer opportunities and challenges for the health and well-being of community members. While participants in each community defined the transect in advance, weather and health concerns required that community members and researchers drive the pre-determined routes rather than travel by foot. Accompanying semi-structured qualitative interviews were conducted with adults in each community, which covered topics such as health and well-being priorities, coping strategies, family and community support, and the impact of economic cycles associated with the oil and gas industry. For example, questions included: (1) What are significant personal community places for your everyday life and resilience, today and in the past? (2) What would you consider as a risky place in your community? (3) What are community places that have significantly changed since you can remember?

#### Ecological and Social-Ecological Resilience

Resilience represents a critical nexus in the field of ecology and our understanding of stable ecosystems. Initially drawing on evidence from predator-prey relationships, Holling (1973) described how ecosystems absorb disturbances and persist in a given state, defined by their structure and function. While systems show, however, a tendency to reach homeostasis, resilience does not depend upon any single regime of behavior and does not always return a system to previous patterns of functioning. A broader understanding of ecological resilience has challenged how we understand ecosystems under stress and anthropocentric bias toward maximum sustainable yields (Gunderson et al., 2012). As we come to understand ecological resilience better, a growing number of scientists are focused on social-ecological resilience to account for the inclusion of human-made stressors on ecological systems and the way different regimes are more or less adaptive for the people who interact with them (Resilience Alliance, 2010; Biggs et al., 2012; Quinlan et al., 2016).

The exploration of social-ecological system adaptation and transformation processes (e.g., protective processes that create system-wide resilience) has shown empirical support for a humans-in-nature perspective (Carpenter et al., 1999, 2001; Folke, 2006; Berkes et al., 2008). Increasingly, social ecologists are developing multiple evidence bases that include traditional knowledge and Indigenous ways of knowing to effectively document changes in social-ecological resilience (Tengö et al., 2014).

Accordingly, to assess ecological resilience, we employed a series of six participatory methods during a multi-day workshop in both the Canadian and South African sites, as is reported in Ungar et al. (2020). The first participatory activity was a Q methodology (Parkins et al., 2015; Pike et al., 2015), an approach to better understand values and perceptions of social-ecological systems and their interactions, and to understand how young people see themselves in connection with place over time, whether in positive, negative, or value-neutral ways (Ungar et al., 2020). The second activity was asset mapping based on a visual approach developed by the Global Ecovillage Network (GEN) to teach the fundamental principles of sustainability and design (Gaia Education, 2012). Cards representing the categories of social, cultural, economic, ecological, and whole systems were displayed in a circle on the floor. Youth were invited to walk around the circle, and both reflect upon and discuss their place-specific experiences related to each card. They then noted their perceptions of community assets, needs, and wellbeing on blank cue cards that were placed on the floor next to each card, which were then charted to determine the subdomains and categories of highest priority. The third participatory activity involved the use of the Environmental Research Institute’s (ESRI) Citizen Science Survey 123, a digital tablet-interface with a Global Information System (GIS) platform. Using the survey, youth were prompted to answer pre-defined questions based on their experience regarding land use impacts, drivers, and pressures, the role of industry (e.g., oil and gas, forestry, agriculture), ecosystem services and supports, and environmental risks. Survey 123 also integrates participatory photography, where youth photographically captured social and ecological relationships within their community, as well as their predictions for desirable and undesirable change in the future.

A fourth activity, Participatory Mapping (McRuer and Zethelius, 2017; Moore et al., 2017; Plieninger et al., 2018), was used to examine youth perspectives of interconnected community places and changing social-ecological systems interactions. Laminated spatial maps on land use, oil and gas infrastructure, green spaces, historical land use changes, and water catchment areas were created based on secondary data available from government databases. Young people worked in small groups to artfully depict their responses to questions concerning their favorite and least favorite environmental places, natural and built environment vulnerability, and disaster mitigation and preferred governance structures. During the workshops, young people were also introduced to ecological monitoring techniques to track air and water quality in their communities, an experiential learning opportunity to investigate community places identified by youth during the participatory mapping. The collected data were used to stimulate reflection on these vital ecological resources, and their significance to wellbeing (Chandler et al., 2017).

The monitoring measures and field equipment used were chosen for their educational potential, as well as their appropriateness of use in each study community, their availability, affordability, reliability, and safety (Liu et al., 2020). Water quality in Drayton Valley, for instance, was monitored using the YSI Multiparameter with Quatro Cable to measure pH, conductivity, dissolved oxygen, total hardness, and temperature; and a YSI 9500 photometer was used with ammonia, chloride, dissolved O_2_, pH, nitrite, sulfate, salinity, phosphorus, and temperature reagents (Liu et al., 2020). In South Africa, the Somerset Educational (Pty) Limited Microlife Water Quality Testing Kit was used to monitor temperature, pH, coliform bacteria, dissolved O_2_ and bio-dissolved O_2_, hardness, nitrites, nitrates, chlorine, and turbidity (Liu et al., 2020). Data collection in both sites was facilitated using Lamotte Insta-Test Natural Water 5-Way Test Strips to study nitrate, nitrite, pH, alkalinity, and total hardness. Monitoring benthic macro invertebrates (i.e., those visible to the naked eye) was also a method employed to assess the biological health of waterways (Liu et al., 2020). Daily air quality (i.e., particulates of 2.5 ppm) was sampled in both research sites using PurpleAir monitoring sensors, and in the Canadian site, daily changes in air quality (i.e., particulate, humidity, and temperature) were collected through Airbeam Air Monitor kits (Liu et al., 2020).

Finally, youth participants in the Canadian community also received certified training in the use of UAVs, or drones, to capture spatial images of their communities. This course was a means to further engage youth in thinking about their place relationships from a novel vantage point, while also providing them with new skills to build their capacity as young professionals (Tabor and Hewson, 2018). Canadian participants used the drones to visually document land use patterns in unpopulated areas of their wider community, then used the images in knowledge sharing activities to highlight changes to the local landscape.

## (Anticipated) Results

**FIGURE 4:**
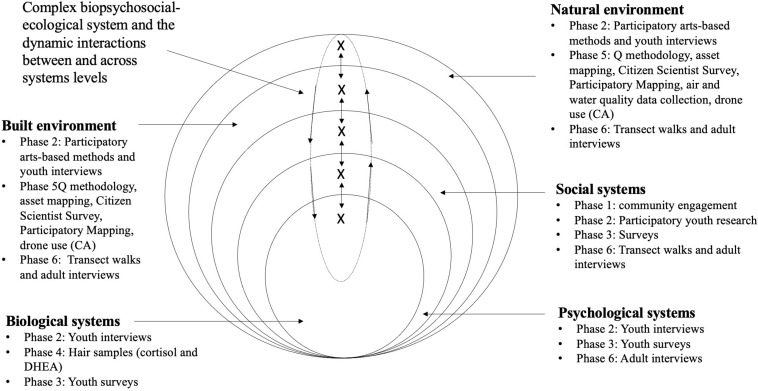
A dynamic multisystemic model of resilience (adapted from Ungar and Theron, 2020). The Xs represent promotive and protective factors at different scales, gathered into a single multilevel system symbolized by the dashed ellipse. The arrows represent the bidirectional influence that PPFPs at adjacent systems levels can have on one another, as well as the relationship across systems levels. Listed under each systems level are the corresponding data collection methods for RYSE.

Data collection and analysis for all phases are ongoing. However, several subsets of the data collected to date have been analyzed, providing clues to how a larger-scale analysis will show the dynamic and multidirectional relationships between risks and PPFPs at each systemic level and between levels. Analyses to date have centered on Phase 2 (youth interviews), Phase 5 (ecological data collection), and Phase 6 (adult interviews) data. The qualitative data analyses thus far have included a narrative analysis of the adult interviews in Phase 6 (Polkinghorne, 1995; McCance et al., 2001; Andrews et al., 2017), as well as a thematic analysis of youth interviews (Braun and Clarke, 2006). While a more comprehensive analysis across the various data sources is pending, from these initial analyses of individual phases we have derived preliminary results denoting the interrelationship between risks and PPFPs at multiple systems levels (see Figure 4 for a visual depiction of this relationship; for further detail about these findings, including the limitations of the analyses employed, please refer to the respective publication).

### Psychological and Social Systems

One-on-one interviews with Canadian youth in Phase 2 revealed that youth exhibited a strong degree of self-regulation when faced with adversity, either by exercising self-efficacy by “working through it” [Participant 004], by being able to “just look at the good things” [Participant 009] or “just figure it out” [Participant 034]. This strong degree of independence when overcoming challenges may in part be fueled by the competitive, industrial nature of the oil and gas industry, which has a profound impact on the community. Yet despite an air of individualism, youth also depend on the support of family and friends during challenging times:

Once things go bad your real friends will be beside you help you through it. I know I have a lot of friends who will help me out. I get depressed really easy and I have suicidal tendencies every once in a while so I have friends for me they play a huge role for me cause they’re there making me smile when I don’t want to and protecting me from all the bad things [Participant 018, youth interview, Phase 2].

There was also a significant cascade of effects across psychological and social systems. Notably, having strong social connections with family and friends often strengthened youth’s internal resources to thrive in challenging times. For example:

I have a good support system, like my family is very supportive, my friends are very supportive, um, I have a really big family just cause my parents are divorced and remarried so it’s like I have, all these people to pull from, um, [long pause] and my mom always taught us to be very independent as well so I’ve got a good skill set that way. I think, so it…it’s good when you’re independent you also do these things, you’re able to tackle things, but when you also have a team, I think that really helps [Participant 047, youth interview, Phase 2].

In contrast, social systems were, at times, contributors to poor resilience at the psychological level, a finding we expect to be reflected also in the biological data. One youth participant lamented that failures in the social systems upon which she relied, challenged her resources at the psychological level:

There’s things [*sic*] that motivate me to be resilient and being rejected for welfare is not encouraging me keep going after the government it’s actually encouraging me to pursue sex work at this point, because that’s I’m faced with not being able to pay my rent and I’ve tried everything and I’m taking a look at my options and I feel like the government will vilify someone like me and will say I’m stupid for choosing these options when they’ve pretty much cornered me into doing it. Say that’s an easy cop out to blame the government but I’ve tried to get an education, I’ve tried to find education outside of school systems that really oppress anyone of different ways of thinking, on top of that as an adult I’ve tried to work a full time job… I just feel like the government because they don’t want to give financial support to the wrong people it’s just shutting out so many people and really discouraging them and making them feel like they’re not good enough or making them to turn to these illegal ways of making money [Participant 001, youth interview, Phase 2].

### Natural Environment

Unfortunately, as per our data on social-ecological systems changes pertaining to land cover and land use change in Phase 5, there has been a notable decrease in the quality of the natural environment, specifically wetlands, barren areas and forests, and instead an increase of built-up and agricultural land (Liu et al., 2020). With oil prices used as the proxy measure for the economic condition, the correlation coefficients between oil price and ecological variables are as follows: wetland and barren areas (−0.59), area of forest (−0.93), area of built-up land (0.68), and area of agriculture (0.77) (Liu et al., 2020). While not conclusive, this points to a decrease in resources that promote resilience at the level of the natural environment.

### Socio-Economic Influences on Social and Psychological Systems

The average household income in Drayton Valley is also strongly correlated with oil price (correlation coefficient = 0.73) (Liu et al., 2020). Debt service ratio at the household level, for instance, is a measure of the household’s ability to produce adequate income to cover its debt payments, and can thus serve as an indicator of the stress level that directly relates to the wellbeing of household members (Statistics Canada, 2017). A correlation coefficient of 0.78 between oil price and household debt service ratio indicates that oil prices positively correlate with individual debt level, implying that people tend to have more debt as the oil economy booms (Liu et al., 2020). Greater loan risks make these individuals more likely to suffer from stress when the oil economy experiences a downturn (Liu et al., 2020).

This vulnerability to hardship during economic bust cycles is reflected in both the youth and adult interviews as well (Phase 2 and 5, respectively) (Theron et al., 2020; Theron and Ungar, in press). Both adults and youth have highlighted the individual- and community-level challenges that ensue during an oil and gas bust, drawing attention to higher crime rates and increases in substance use, and the need to adapt to these challenges:

I mean my dad used to be an alcoholic so I guess. When his job took a huge turn and like when I got sick that was a big stopping point for him. Then he stopped drinking but he definitely uses it to cope a whole bunch especially in 2011 when the economy was a bad year. A lot of my friends turned to drugs in high school just for that. A distraction, I dunno about a coping mechanism but a distraction for everything that’s going on in their lives for sure. Cause a lot of them like I said didn’t have the best family homes right. None personally, cause I was lucky in the fact that my mom was there and my family was there [P 35, adult interview, Phase 6].

The above quote, while illustrative of how the social conditions within which community members live and work can serve as risk factors for their well-being, also points to how a supportive family life can serve as a promotive factor for one’s resilience, and thus, a protective factor for their individual well-being. And indeed, while the survey data has yet to be analyzed in full, a descriptive analysis of the T_1_ data revealed that 23% of respondents experienced family issues (e.g., the death of a parent/caregiver, parents/caregivers separating, and experiencing severe mental and physical health issues), while 71% reported having strong parental support (i.e., a feeling of trust, being looked out for, and feeling comfortable discussing challenges with parents). It is through the interrelationship of these various risks and PPFPs at various systems levels that we are gaining an understanding of the dynamic interactions between systems.

### Further Analyses

In addition to ongoing qualitative analyses, we anticipate further analyses using various subsets of the collected data to understand how risk and resilience interact at different levels in these settings. For example, while studies have individually examined the importance of PPFPs like social support and neighborhood perceptions on important outcomes like wellbeing and mental health, it is unclear how such factors may interact or produce cumulative effects. Network analysis is a technique which is beginning to be used in studies of resilience (Fritz et al., 2018b; Höltge et al., in press) and can help to identify how different PPFPs at different levels (e.g., family support, community belonging, proximity to green spaces) may connect with each other as well as risk factors (e.g., family adversity) and important outcomes (mental health, school/work engagement).

The collection of longitudinal data means we are also able to analyze how PPFPs influence each other over time and potentially contribute to developmental processes. For instance, regressing PPFPs, risk factors, and baseline outcomes on outcomes measured at the final timepoint allows for the identification of important predictors as well as their relative contribution. Similarly, given that resilience trajectories can vary (e.g., see Bonanno and Diminich, 2013), latent transition analysis may be employed to determine different the risks and PFPPs associated with individuals who appear to cluster into distinct patterns within outcomes (e.g., minimal impact, recovery).

## Discussion

If resilience is to be understood as a multisystemic process, encompassing biological, social, institutional and ecological factors that are mutually co-dependent, then research methods are required that capture data across multiple systems within the same study in ways that are reasonably accessible to researchers with limited expertise in more than one discipline. While the RYSE project has been an ambitious and well-funded project, it has been challenging to find ways to effectively assess the resilience of different systems in the same study. While no single assessment is comprehensive, combined, all six phases of the research are helping to describe the complexity of resilience-related processes for young people in a changing economy that is becoming more stressed as oil and gas prices decline. In developing this methodology, we experienced a number of challenges:

1. To study resilience multisystemically required working with a multidisciplinary team of researchers and postdoctoral fellows with complementary areas of expertise and an openness to looking for linkages between bodies of theory. Accomplishing this has meant team members working outside their intellectual comfort zones and engaging in scientific methods that are less familiar, posing what have been recognized as ‘cognitive obstacles’ in interdisciplinary research [a challenge that has been recognized elsewhere as ‘domain specificity’ (MacLeod, 2018)].
2. While our two research sites are dependent upon the oil and gas industry and thus community members share a number of realities (e.g., economic precarity, environmental concerns, etc.), studying the multiple systems implicit in resilience across these sites was not always an entirely parallel process. Instead, the contextual dynamics of each community shaped the methodology in each site, for instance by having LACs determine the inclusion and exclusion of specific survey items, omitting the use of drones in the South Africa site amidst concerns for the safety of youth researchers, the complexities of sampling and assaying African hair, and other such concerns. For instance, many young African men prefer to have very short hairstyles. This preference complicated analyses, given the importance of the length of a hair sample (Greff et al., 2019). Similarly, many young African women wear braids or weave, and this complicates sampling of their natural hair (Manns-James and Neal-Barnett, 2019). In response, the local laboratory suggested that hair samples be collected according to weight (rather than length). They also taught researchers to sample young women’s hair without damage to braids or weave. This speaks to the importance of studying multi-systemic resilience in context-appropriate ways and to engaging local experts (e.g., the University of Cape Town Hair and Skin Laboratory for the assaying of the SA hair samples).
3. Deciding which systemic levels to focus attention on was a balance between being theoretically sound and keeping the research feasible. For example, blood samples would have been a better biological marker for stress hormones and expanded the scope of the analyses but was too costly and impractical, especially in South Africa where there are strict protocols for working with any biological samples drawn from humans.
4. Assessing the ecological conditions in communities dependent on oil and gas industries can be perceived as politically threatening. We therefore emphasized the role of citizen scientists and restricted our data collection to relatively benign measures like water and air quality, or easy to measure environmental markers like acreage under cultivation and green spaces. Though broader and more sophisticated measures of ecological resilience would have been useful, the nature of the study required a level of community engagement that demanded careful negotiation with stakeholders and attention to social and political exigencies.
5. There was a need to communicate the purpose of the study and its design to key stakeholders in the community, from members of the town council to educators, parents and young people themselves. Carrying on years of complex assessments with very large numbers of people in small towns can cause some concern. Investment in knowledge mobilization activities to share results incrementally and ensuring a community presence for the study at annual events has helped maintain participant trust and engagement. Examples of such knowledge mobilization events include certificate ceremonies for youth engaged in the participatory research components, youth showcase events to share results with the wider communities, youth presentations to oil and gas companies (e.g., SASOL in South Africa), and newsletters and other materials to promote the project within each community. Some of these knowledge mobilization initiatives have been documented in local news outlets, for instance by Mathebula (2019), and Brazeau Empowerment Support Team (2020). We are also in the process of engaging youth to lead end-of project knowledge mobilization initiatives such as presentations to Town Council, oil and gas companies, and other potential end-users; as well as the creation of videos, policy-briefs, or actionable projects that promote awareness and uptake of the data while also building youth capacity.
6. Keeping the focus on resilience has meant shifting the conversation to positive adaptations rather than individual and community vulnerabilities. By its nature, resilience research asks a different set of questions about human development. For both communities, there was a tendency to want to discuss problems rather than to look for promotive and protective processes already operating in both research sites.
7. Analysis of the data has proven demanding. While there is support methodologically to analyze and publish data from a single system (e.g., the results of the longitudinal survey, or the ecological data), we have had to be more creative studying the effects of the resilience of one system on another. Our approach has been an incremental production of results, with papers now appearing that detail patterns of resilience at different systemic levels, or combinations of two systems (see Theron et al., 2020; Twum-Antwi et al., 2020; Ungar and Theron, 2020; Theron and Ungar, in press). Forthcoming papers will use techniques like Network Analysis (Fritz et al., 2018b) and variations of Grounded Theory (Glaser, 1998; Charmaz, 2006) to integrate data sources and facilitate multisystemic accounts of resilience.

## Conclusion

While the methodologies to understand resilience within and between systems are still emerging, there is a clear interest in the need to account for the complexity of the interactions that change a system’s regime of behavior for the better. To consider the resilience of a single human system in isolation from biological, social, and even economic processes is no longer tenable as good science. The microcosm of an oil and gas dependent community illustrates our point. Individual health will be affected by relational factors, themselves stressed by changing economic conditions that are far beyond the power of local changemakers to influence. Thriving during a period of industrial and economic disruption will require resilience of multiple systems triggering positive adaptations simultaneously at different levels. If we are to understand these changes, we will need more comprehensive, multisystemic methodologies to study resilience.

## Data Availability Statement

The raw data supporting the conclusions of this article will be made available by the authors upon request, without undue reservation.

## Ethics Statement

The studies involving human participants were reviewed and approved by Dalhousie University Health Sciences Research Ethics Board and the University of Pretoria Research Ethics Committee. Written informed consent to participate in this study was obtained for all participants. Where participants were younger than 16 in Canada, or 18 in South Africa, consent was obtained from the participants’ legal guardian/next of kin.

## Author Contributions

MU and LT conceived the idea for this study, collaboratively designed, and directed each phase of the project in their respective research sites (MU in Canada and LT in South Africa). MU led in the drafting of this manuscript with the close support and conceptual contributions of LT. KM significantly contributed to manuscript writing, the qualitative components of the analysis, and pulling the various pieces of the project together. PJ also contributed to the manuscript writing and providing critical revisions, as well as leading much of the quantitative design and data analyses over the course of the study. All authors read and approved the final version of the manuscript.

## Conflict of Interest

The authors declare that the research was conducted in the absence of any commercial or financial relationships that could be construed as a potential conflict of interest.
